# Parasite hideouts: Preserving native cavity-nesting Hymenoptera through pollinator hotel management

**DOI:** 10.1371/journal.pone.0357475

**Published:** 2026-10-06

**Authors:** Bailey B. Lankford, Maria Escobedo, Sylvia Yoo, Elsa Youngsteadt, Denis S. Willett, Camila C. Filgueiras

**Affiliations:** 1 Department of Biology, University of North Carolina Asheville, Asheville, North Carolina, United States of America; 2 Department of Applied Ecology, North Carolina State University, Raleigh, North Carolina, United States of America; University of Carthage, TUNISIA

## Abstract

As many pollinator populations around the world decline due to habitat loss, improving management practices for native pollinators becomes a priority. Pollinators are essential to healthy ecosystems and play irreplaceable roles in the global food system. Pollinator hotels can provide a safe, protected space suited for many different species, and can also be used as educational tools. However, these structures also often contain parasites attracted to the nests within, which can multiply without maintenance. Pollinator hotels are usually filled with nesting cavities in a variety of materials including cardboard, reeds, bamboo, and native plant stems. This study sought to determine whether any materials are more or less prone to parasitic infestation and whether introducing emergence boxes for overwintering bees could offer a solution. We first conducted a family-level biodiversity survey to examine the parasite populations in pollinator hotels. Second, we conducted a controlled study of parasite burden in three material types. Third, we investigated emergence boxes as a tool to facilitate cleaning and maintenance of pollinator hotels with the overall goal of reducing parasitic infestation. We found that cardboard tubes had far higher rates of parasitism than other materials across multiple families of parasites. Elimination of cardboard nest tubes, coupled with regular maintenance, may help protect against parasitism. We also found emergence boxes to be a safe and effective way to house overwintering bees while maintenance is performed; in emergence boxes, very few new parasite holes appeared, and bees and wasps rarely constructed new nests. These results provide guidance for researchers and educators constructing pollinator hotels to select nest materials that safeguard pollinators and implement evidence-based maintenance practices that support pollinators across a wide variety of ecosystems.

## Introduction

Pollination, and more specifically insect pollinators, are essential to healthy ecosystems and play a critical role in both crop production and the reproduction of many flowering plant species. Nearly 70% of leading crop species experience increased production when pollinators are present [1]. The European honeybee (*Apis mellifera*) often receives the most attention and recognition as a pollinator. However, *A. mellifera* is only one of over 20,000 species of bee documented worldwide [2]. In contrast, other species, such as bumble bees, mining bees, mason bees, and leafcutter bees, are under-studied and under-appreciated despite evidence that native pollinators such as these are more effective than honey bees at pollinating a number of flowering crop species [3–5]. Native solitary bees such as *Osmia lignaria* have even been developed as managed crop pollinators in North America [6,7]. These bees also exhibit a diverse array of life histories, ecological niches, and nesting habits. The majority (roughly 90%) of bee species are solitary [8] and build nests in soil (ground nesters) or dead wood, stems, or piles of brush (cavity nesters) [9]. While some native bees are generalists, gathering resources from a wide array of flowering plant species, some are specialists, and will only collect pollen from a select few species [9]. This intricate dependence on specific plant species and habitat structures makes native pollinators vulnerable to habitat loss and human disturbance [9].

One increasingly popular tool to help mitigate these problems for native pollinators is the pollinator hotel. While the majority of solitary bee species are ground nesters (approximately 70%), pollinator hotels can serve as habitat for the remaining 30% of cavity nesting species [10]. These structures are also commonly called bee hotels, but they often host a variety of solitary wasps as well as cavity-nesting bees [11]. Mason bees (*Osmia* spp.) and leafcutter bees (*Megachile* spp.) are the two most common genera reported utilizing pollinator hotels [11]. Mason wasps (Vespidae: *Eumeninae*), grass carrying wasps (*Isodontia* spp.), and square-headed wasps (*Crabronidae*) are also frequently observed inhabiting the hotels [9]. While bee species provide valuable pollination services, solitary wasps found in pollinator hotels also contribute to controlling pest insect populations [12].

The popularity of pollinator hotels is also at least partially due to the hotels’ potential as an educational tool. Pollinator hotels are conspicuous structures, and often placed near gardens in prominent locations visible to the passing public. In botanical gardens or other official public spaces, there are usually also educational signs posted nearby with information about what the pollinator hotels are, what kinds of insects can be found inside, and what these animals contribute to the wider ecosystem. Building and installing pollinator hotels has been an integral part of many pollinator education campaigns and citizen science projects, and the hotels themselves provide a venue for people to observe insects that they might not otherwise notice [9,13].

Pollinator hotels replicate cavity nesting species’ natural nesting sites by offering access to hollow tubes, often made of bamboo, reeds, cardboard, drilled wooden blocks, or the stems of native plants [9]. Providing the bees with a diverse array of materials may help to attract a wider range of bee species [14]. However, each material type has its own unique attractants and drawbacks. Drilled blocks, for example, are known to be extremely attractive to both bee and wasp species but are difficult to maintain [15]. Any old nests left in the blocks may contain diseases or parasites that are impossible to completely remove. Likewise, positioning the drilled holes too near to the edges of the block can make the nests susceptible to parasitism, especially by the *Leucospis affinis* wasp [16]. Bamboo or reed tubes with too large a diameter may be either left completely unoccupied or preferentially host large non-native bees such as the aggressive giant resin bee (*Megachile sculpturalis*) [14]. Cardboard tubes are commonly available in commercial nest kits, but very high rates of parasitism have been reported [17].

Because of these challenges, and especially the risk of parasitism, it is paramount that pollinator hotel installation follow known best practices. Some of these techniques are well supported and data-driven; for example, there is empirical evidence to support limiting the size of pollinator hotels to about 100 nest tunnels [18]. The diameter and length of nest tunnels should also be tailored to the habits of bees known to nest in the area, as tunnels with diameters narrower than a species’ ideal may produce smaller offspring and a sex ratio skewed towards males [19–21]. Successful pollinator hotels are also located close to sources of pollen and nectar, as many small solitary bee species have limited flight ranges [21].

Regular maintenance is also essential. Many pollinator hotel maintenance practices have extensive anecdotal evidence, but are not backed by empirical data. For example, emergence boxes are small, mostly enclosed containers in which nest materials can be placed to provide a transitional area where the bees can emerge [9]. The old nesting materials can then be removed and cleaned over the winter while the bees are inactive. In the summer, the bees emerge and are free to make nests in fresh materials in the main hotel. These boxes often come with commercial pollinator hotel kits, but there is little to no empirical evidence that demonstrates whether the boxes are an effective tool for pollinator hotel maintenance. There are risks that creating an even greater concentration of nest sites within the emergence boxes can encourage parasitism, or that the emerging insects will return to nest in the boxes rather than the newly-cleaned hotels. This lack of data matters because cleaning pollinator hotels is an essential maintenance task. Chalkbrood and parasitic mites can linger in old nests and spread throughout the pollinator hotel without regular cleaning [17]. Other parasites, such as Houdini flies, beetle larvae, and parasitoid wasps may also accumulate in pollinator hotels without regular maintenance [17].

Parasitic and parasitoid wasps are a common secondary inhabitant of pollinator hotels [9,17,22,23]. The most common of these are *Monodontomerus* species (Torymidae), *L. affinis* (Leucospidae), and members of the family *Chrysididae*. *Monodontomerus* wasps are tiny parasitoids that lay their eggs in *Osmia* and *Megachile* nests [17]. *Leucospis affinis* also parasitizes the larvae of *Osmia* and *Megachile*, along with some species of solitary wasps [22]. Members of the *Chrysididae* family are common kleptoparasites of several pollinator hotel residents [24,25]. There is some indication that parasitism may be more common in pollinator hotels than in dispersed nesting sites, and that this increase may correlate with the higher density of nest sites that pollinator hotels encourage [13,26,27]. Parasitism in pollinator hotels can disproportionately affect the native bee residents of the hotel, rather than introduced bees or either native or introduced wasp species [13]. If left completely unmanaged, pollinator hotels may become more of a liability to their native, solitary inhabitants [13]. To be effective tools, pollinator hotels must be installed and maintained on a regular schedule, using tools to both preserve the native bee populations and reduce the burden of parasites and parasitoids.

In this study, we investigated the parasite burden present in four pollinator hotels. We also conducted the first empirical study on whether emergence boxes could be implemented as an effective maintenance and cleaning tool. We first conducted a biodiversity study of a single pollinator hotel in North Carolina to understand inhabitant profiles. Secondly, we surveyed the parasite burden across four pollinator hotels. We hypothesized that the parasite burden would be highest in cardboard nest tubes as previously described, but that nests made of reeds would also have a higher parasite load than bamboo tubes because of the relative thinness of the reed tube walls. Lastly, we tested the efficacy of emergence boxes as a maintenance tool. Specifically, we evaluated whether nests placed in emergence boxes would accumulate futher parasitism and whether bees and wasps would construct new nests inside the old materials in the boxes. In half of the emergence boxes, we implemented a one-way valve that we hypothesized would further reduce the rate of bees re-entering the emergence boxes.

## Materials and methods

No specific permits were required for sample collection in these studies. All samples were collected with permission from either the University of North Carolina Asheville or North Carolina State University. No protected or endangered species were collected.

Three studies were conducted on a total of four pollinator hotels between the summer of 2022 and the spring of 2024. Study 1, conducted in the summer of 2022, concerned only the pollinator hotel located on the University of North Carolina Asheville campus, and examined the family-level diversity present within the hotel.

Studies 2 and 3 were conducted concurrently from February 2023 to March 2024 on both the UNC Asheville hotel and three smaller pollinator hotels on the campus of North Carolina State University in Raleigh, North Carolina (Table 1). These three hotels were denoted Centennial, Court of the Carolinas, and Kilgore, based on the area of NC State Campus in which they were located. All four pollinator hotels were located in similar semi-urban settings near areas of frequent travel, had all been established for several years prior to the study, and contained cardboard tubes, reeds, bamboo, and wooden blocks for the insects to nest in. The UNC Asheville pollinator hotel also utilized hollow sticks, drilled logs, and clay blocks as nesting materials, but these three material types were excluded from all but the biodiversity survey due to lack of observed nests and continuity with the other three hotels.

**Table 1:** Local information and emergence box treatments (Study 3) present at the pollinator hotels. Distance from garden refers to the space between the hotels and the associated gardens.

Name of Hotel	Coordinates	Treatment (Emergence Box)	Distance from Garden (m)	Notable Flowering Plant Species
UNCA	35.617N, 82.563W	Control, Valve	1	*Solanum carolinense*, *Rudbeckia hirta*, *Lactuca* spp., *Coreopsis* spp., *Salvia lyrata*, *Helianthus angustifolius*, *Geranium* spp., etc.
Centennial	35.766N, 78.676W	Control, Valve	2–3	*Callicarpa americana*, *Salvia* spp., *Helianthus angustifolius*
Court of Carolinas	35.786N, 78.666W	Control, Half-valve	2–3	*Narcissus* spp., Asteraceae spp.
Kilgore	35.788N, 78.672W	Control, Half-valve	1.5–2	*Cercis canadensis*, *Hibiscus* spp., *Salvia* spp., Asteraceae spp., *Ipheion uniflorum*

Each of the four hotels was located near at least one maintained garden containing multiple flowering plant species (Table 1). While complete lists of flowering plants present in each garden were unavailable for the duration of the studies, the most prominent species observed during the collection period of studies 2 and 3 are listed in Table 1. The three NC State hotels were all small and uniform in size, and positioned on posts approximately a meter off the ground. The UNC Asheville hotel was much larger in size, and positioned approximately six inches from the ground. All four pollinator hotels were oriented to face either the southeast or southwest, which is consistent with best practices for pollinator hotel installation in the northern hemisphere [9]. The UNC Asheville and Court of the Carolinas hotels were mostly in full sun, but had periods of partial shade. The Centennial and Kilgore hotels experienced full sun.

### Study 1: Biodiversity survey

#### Site characteristics.

The pollinator hotel located on the campus of the University of North Carolina Asheville was established in 2016. It is located in a semi-urban environment approximately 5 meters from areas of high foot traffic. The hotel is also between 1 and 5 meters from two separate pollinator gardens that contain a range of native flowering plant species, and it is backed by a small, wooded area (Table 1). The hotel had not been ideally maintained for several years before the date of this study. Before beginning collections, the pollinator hotel was divided into twelve equally sized boxes in three rows of four, numbered one through twelve from the top right to the bottom left (Fig 1). These subsections were created to ensure equivalent sampling across the pollinator hotel. All samples gathered were kept separated by box number.

**Fig 1:**
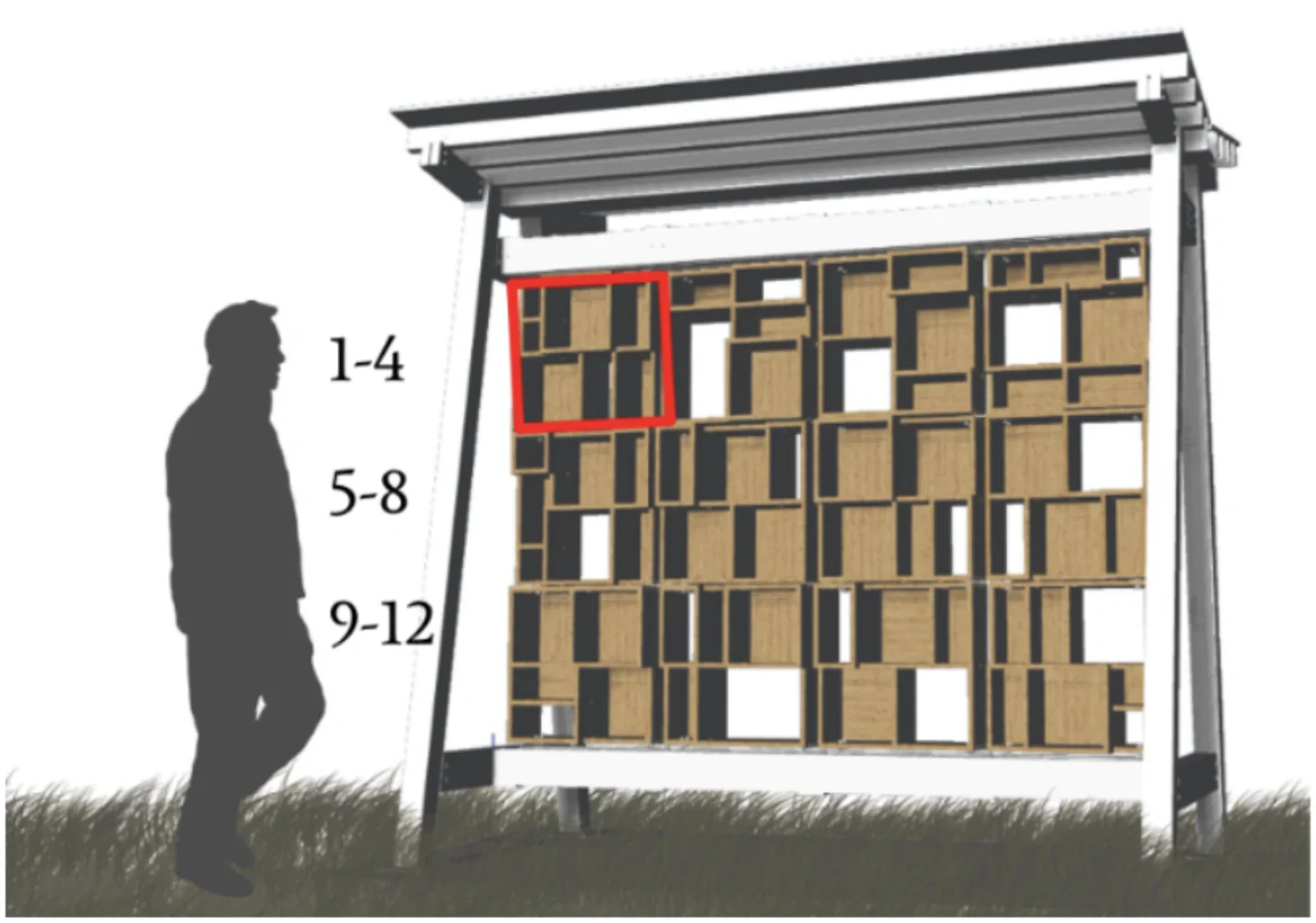
Model of the UNC Asheville pollinator hotel drawn to scale. This pollinator hotel was subdivided into twelve equally sized boxes for the purpose of these studies. The first box is outlined in red.

#### Sample collection and analysis.

Adult insects were randomly collected from in and around the pollinator hotel using aspirators. These collections were done on three separate days in June, July, and August 2022. Adult insects from different boxes within the hotel were collected in separate aspirators, euthanized separately using 95% ethanol vapor, and stored frozen in separate containers. All adult insects were thawed, pinned, and identified to family using a dissection scope and the dichotomous key from Borror and Delong’s *Introduction to the Study of Insects* (7^th^ edition, 2004).

On a separate day in July, nesting materials were randomly collected from each of the twelve boxes within the hotel (Fig 1). This pollinator hotel utilized paper tubes, reeds, hollow sticks, bamboo, drilled logs, and clay blocks as nesting sites. Clay blocks were later excluded from the sample as there were no nest sites evident in any of the clay samples collected. The types of nest materials were not distributed equally across all twelve boxes, but a sample of every material present in each box was collected. In total, between five and twenty nest tubes were gathered from each box and frozen. These nests were opened using a hobby knife to make a small, precise cut at the front end. Paper tubes were then unwound along the seam, and reeds and bamboo were carefully cracked open. All adults found within these nests were pinned and identified to family using the same dichotomous key.

#### Data analysis.

All data were analyzed using the R Statistical Programming Language (version 4.5.1) and RStudio (version 2025.09.1 + 401) [28,29].

To visualize variation in insect abundance and family-level richness across the 12 boxes, heatmaps were created by transforming the initial raw count and family number dataframes into matrices, and assigning the values to a color ramp. A chi-square test of independence was conducted to examine non-random distribution of insects. The total count data were then divided by material type (adult insects caught via aspirator while flying around the pollinator hotel were labeled “No Material”), and classified as either parasites or non-parasites.

### Study 2: Parasite abundance and distribution

#### Site characteristics.

This study utilized both the UNC Asheville and NC State pollinator hotels, which were established in 2016 (Table 1). Bundles of new, unused nesting materials were created using rubber bands. Each bundle contained one paper tube with a diameter of 8 mm, one paper tube with a diameter of 6 mm, one paper tube with a diameter of 4 mm, one reed with a diameter of 8 mm, one reed with a diameter of 6 mm, and one stick of bamboo with diameters ranging from 10 mm–6 mm. These diameters were chosen because they are standard in kits available from commercial retailers. Tube diameter varied across material types, but was not acknowledged as a covariate in this study. Therefore, in the results, we cannot completely separate the effects of material and diameter. The paper tubes and reeds were sourced from Crown Bees (Woodinville, WA, United States), while the bamboo was cut from a yard in West Asheville, North Carolina. All materials had one open and one closed end and were between six and eight inches in length.

#### Sample collection and evaluation.

Four to six of the nest material bundles were randomly placed in each of the four pollinator hotels on the second weekend of the month in February, April, and June 2023. The bundles remained in the pollinator hotels for approximately two months and were collected for analysis on the second weekend of April or June, or the third weekend of August, respectively. All bundles were found and removed from the hotels at each collection period with the exception of the June collection from the Centennial pollinator hotel, which had been vandalized.

The samples were moved back to Asheville, North Carolina, and processed within a week of collection. They were kept separated by month, pollinator hotel, and material type (reed, paper tube, or bamboo). Processing involved carefully opening each individual nest site with a hobby knife using the same procedure detailed in the biodiversity survey and analyzing the contents. Any already-hatched insects were euthanized with 95% ethanol and identified to family using the dichotomous key found in Borror and Delong (7^th^ edition). Any intact cocoons were placed into well plates whose lids had been punctured to facilitate airflow. The well plates were kept in an incubator initially set to 1°C to simulate natural winter conditions [30]. At the end of February, the temperature was set to the average of the weekly temperatures of Raleigh and Asheville. Mean monthly temperatures have previously been used successfully for overwintering bees [30]. This incubator also contained a dish of water to increase humidity, as is often recommended for successful overwintering by companies that sell solitary bee cocoons [31].

The incubator was checked every other day for eclosed adults. The rearing period lasted one full year from the start of collection, and concluded in August 2023. All insects that eclosed within the well plates were removed and identified to family, using the dichotomous key noted above. The hatching dates, material type, month, and location of origin of the hatched insect were recorded. Some small parasitic wasps escaped from the well plates through the airflow holes. These insects were collected, euthanized, and identified to family. The date of their escape was also recorded. Due to their extremely small size, eulophid wasps were especially prone to escape. To reduce the risk of cross-contamination with this parasite, any well containing eulophid wasps was immediately taped over.

#### Data analysis.

All data were analyzed using the R Statistical Programming Language (version 4.5.1) and RStudio (version 2025.09.1 + 401) [28,29]. The following packages were also used: *car* [32], *readxl* [33], *tidyverse* [34], and *emmeans* [35]. Raw parasite count numbers were evaluated for effects of family against month, location, and material type using generalized linear models with Poisson distribution, followed by an examination of the pairwise comparisons using *emmeans* [35]. P-values were corrected with the Tukey method. ANOVA tests were also performed to determine the overall significance of these variables and their interactions. All test results were evaluated for significance at α = 0.05.

### Study 3: Emergence boxes

#### Site characteristics.

This experiment used all four pollinator hotels to test the efficacy of emergence boxes as a tool for pollinator hotel maintenance. In February of 2023, 80–85 occupied nest materials were collected from each pollinator hotel. Occupation was determined based on the presence of a cap and the weight of the material as compared to visually empty materials. This array of materials spanned all nest types and diameters present within the individual hotel and were taken from multiple locations. The materials were kept separated by pollinator hotel of origin through this process.

Rectangular overwintering boxes were constructed out of yellow pine (Fig 2). All panels were attached with a nail gun as well as screws and wood glue. A front panel was also attached with slight overhang on the left and right sides, and a hole was drilled in the center with diameter 0.5 inches (Fig 2). The back panel was cut but not attached until all materials were inside. All gaps except the front entry hole were filled in with a mixture of wood glue and sawdust. The tops of these boxes were covered with Vycor Pro Fully Adhered Butyl Flashing Tape (GCP Applied Technologies, Alpharetta, Georgia) to increase waterproofing and prevent rain incursion. A small piece of PVC conduit (CANTEX INC., Fort Worth, Texas) was attached to the outside of the entry hole with hot glue (Fig 2). While all nest boxes had PVC conduit pieces over the entrance, the PVC conduit on the treatment boxes contained a one-way valve apparatus constructed from interlocking false eyelashes (Kiss USA, Port Washington, NY) and attached with hot glue (Fig 3). The eyelashes overlapped so that a bee exiting the emergence box could push through easily, but a bee attempting to enter the box would need to bend the entire valve out of shape. The PVC conduit on the control boxes was left open without a one-way valve.

**Fig 2:**
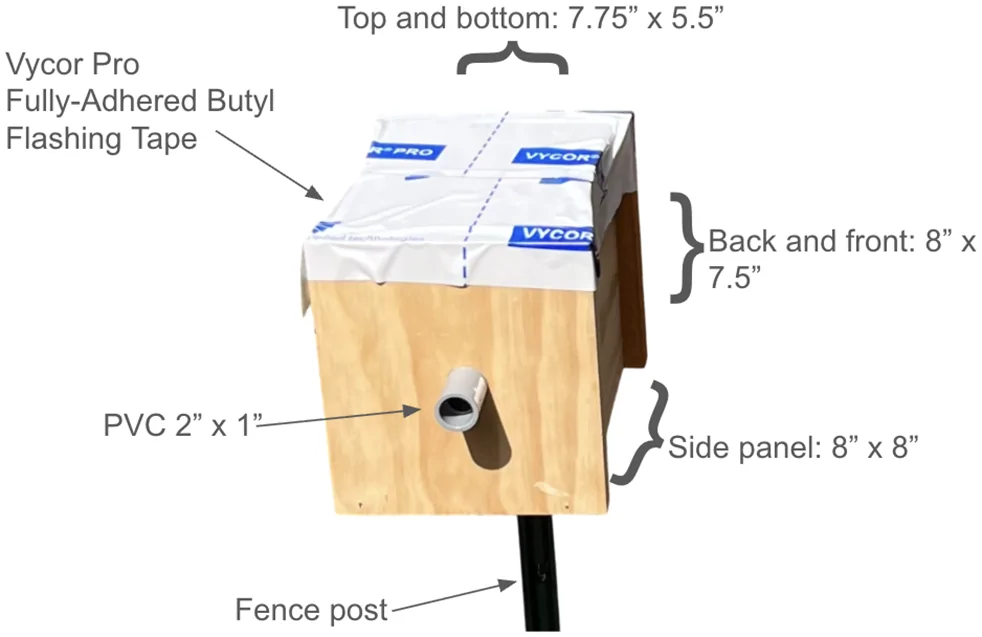
Schematic of overwintering boxes constructed from 1” thick boards of yellow pine.

**Fig 3:**
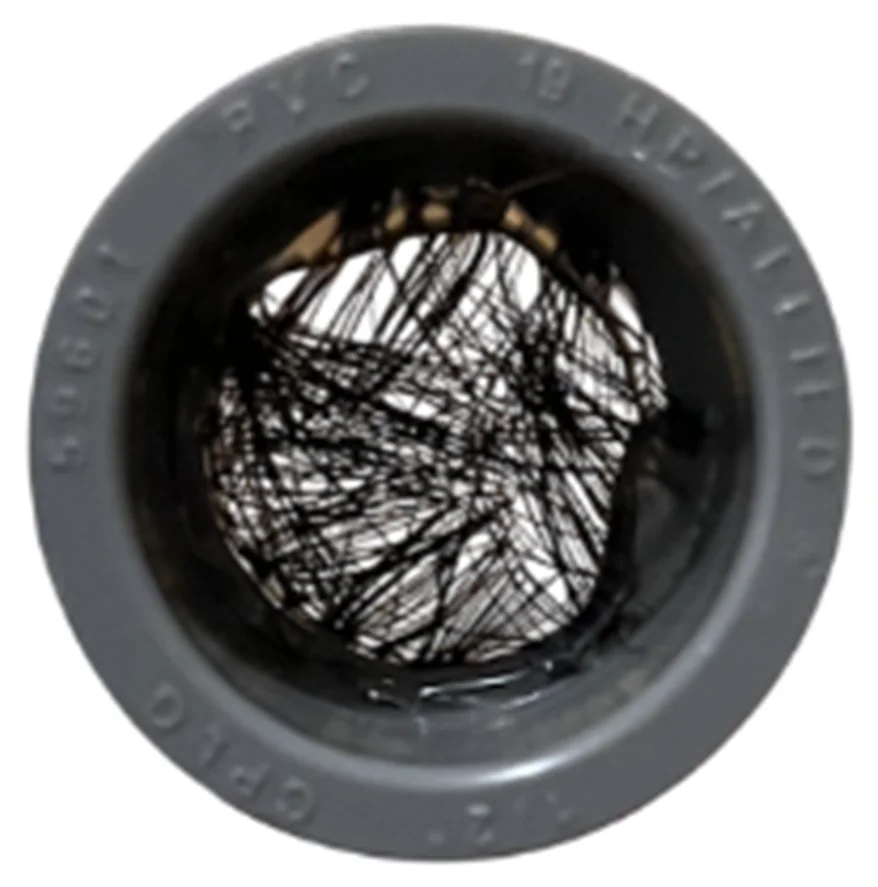
PVC pipe (2” length by 1” diameter) one-way valve constructed from false eyelashes and hot glue.

#### Sample collection and analysis.

Samples were placed into the emergence boxes as whole, unaltered nest tubes, rather than separate cocoons. Prior to placement in boxes, each nest tube was labled with a unique ID and its diameter, material type, number of holes (indicative of previous parasite infestation [36]), nest cap material, and nest cap condtion were recorded. After the emergence boxes were opened in March 2024, the materials were evaluated for new parasite oviposition holes, as well as nest cap presence and material. The presence of any new parasite holes after a year in the emergence box would indicate that parasitic wasps entered nest materials while inside the box. After completing their nests, solitary bees and wasps seal off the nest with a solid cap [9]. Different species use different materials to build these caps; leafcutter bees, for example, use leaf material, while grass wasps stuff strands of grass into the nest opening, and mason bees often seal their nests with mud [9]. When the offspring emerge, they make a hole in the nest cap [9]. One of the principal concerns with the use of emergence boxes was the potential for bees to re-nest inside the boxes rather than making nests in the nearby pollinator hotels. If the material of the cap changed (from mud to leaves, for example) or if the cap initially contained a hole, but was intact after removal from the box, another bee or wasp had likely re-nested in that location. Nest caps were sorted into brown mud, fiber, grey mud, grass, leaf pulp, orange mud, resin, or yellow mud. A total of 326 nest tubes were included in the study. We randomly assigned each nest tube to be either reared in the lab (65), placed in a control box (130 total across 4 boxes), or placed in a treatment box (132 total across 4 boxes). Nests assigned to either the control or treatment box were bound together with rubber bands with a longer stick so that the open ends would not fall against the side of the box and prevent escape. These nests were then sealed inside the overwintering boxes with screws and a piece of wood identical.

One control box and one treatment box were assigned to each pollinator hotel. Two of the treatment emergence boxes (located at Court of the Carolinas and Kilgore) lost their valves approximately halfway through the study period in June. These two emergence boxes have therefore been reclassified as belonging to a third treatment (Half-Valve). After this reclassification, there were four boxes belonging to the Control group, two boxes belonging to the Valve group, and two in the Half-Valve group. All nests were placed inside the overwintering box which corresponded to their pollinator hotel of origin. These overwintering boxes were then placed on metal fence posts approximately one meter off the ground and one to two meters away from each individual pollinator hotel and left in place until March 2024.

To assess the family distribution present in the populations of the pollinator hotels, a small portion of the samples were brought back to the NEMA lab on the UNC Asheville campus to be reared out. These nests were kept separated by the hotel of origin and opened within a week of collection, using the same methodology described in Study 1. Their contents were placed in well plates with holes in the lids for airflow. The well plates were kept in an incubator using the same temperature settings and humidity control as the parasitic contamination study (Study 2). Any insects that eclosed under these conditions were removed from the incubator, euthanized with 95% ethanol vapor, and identified to family using the dichotomous key from Borror and Delong (7^th^ edition). The hotel of origin of each hatched insect was also recorded.

In early March 2024, all overwintering boxes were removed and brought back to the UNC Asheville campus. They were opened within a week of removal, and the contents were analyzed. Any adult insects present within the box were recorded as well as box conditions (mold, debris, presence of spider webs, etc.). All data gathered at the beginning of the study (on nest cap material, holes present in the sides, etc.) were regathered to check for changes that would indicate parasitism or re-nesting. All nest materials within the boxes were then opened, and if any intact cocoons were present, they were removed and reared in well plates in an incubator (same conditions in Study 2).

#### Data analysis.

All data were analyzed using the R Statistical Programming Language (version 4.5.1) and RStudio (version 2025.09.1 + 401) [28,29]. The following packages were also used: *ggplot2* [37], *dplyr* [38], *tidyverse* [34], *ggsignif* [39], *scales* [40], *pscl* [41], *viridis* [42], *lme4* [43], *ggsankey* [44], *ggalluvial* [45], *ggpubr* [46], and *pscl* [41]. Initial count data of parasitoid holes was evaluated against treatment using a zero-inflated negative binomial model in the package *pscl* to handle overdispersion and an overabundance of zero values [43].

To examine whether the proportion of re-nesting events differed among the four hotels or among material types, we ran a logistic regression model with the emergence box number included as a random effect. Chi-square tests were run to determine if specific families were correlated with specific cap or material types, and ANOVA tests were run to determine if re-nesting events differed among bee hotels, material types, or emergence box treatment groups. All test results were evaluated for significance at α = 0.05.

## Results

### Study 1: Biodiversity survey

In total, 10 different Hymenoptera families and over 600 adult insects were seen inhabiting the pollinator hotel during this study. The total number of insects observed in each of the twelve sub-boxes of the pollinator hotel ranged from 3 to 234 (Fig 4). However, all sub-boxes except Box 7 contained between 3 and 100 insects. The outlier in Box 7 (234 insects) was caused by a large nest of Formicidae found inside some of the nest materials (Fig 4). A chi-square test of independence revealed significant non-random distribution of insects across the pollinator hotel ($\chi^{2}=278.64$, df = 6, p<2.2e−16). While the outlier in Box 7 was a major driver of this result, Box 10 also contained significantly more insects than expected, and Boxes 6 and 11 contained fewer than expected by the model. However, there was no overarching pattern in the total numebers of insects across rows or columns of the pollinator hotel. The total number of families found inhabiting each of the sub-boxes ranged from 3 to 8 (Fig 4). Box 7 also contained the joint highest number of families observed, along with Box 5. Although the left of the hotel receives more sunlight, a chi-square test of independence revealed no differences in the distribution of families across the hotel ($\chi^{2}=3.5186$, df = 6, *p* = 0.7415).

**Fig 4:**
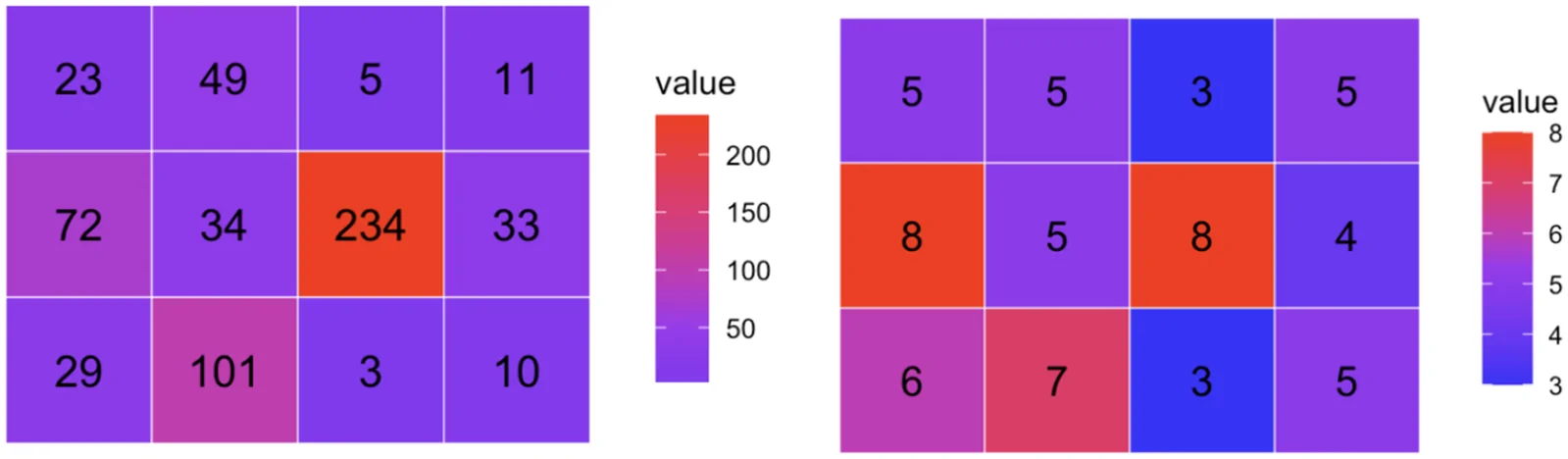
Heatmap of insect abundance and family diversity. Heatmap of the total number of individual insects (left) and of the total number of insect families (right) observed in each of the twelve sub-boxes at the UNC Asheville pollinator hotel. The outlier nest of Formicidae in Box 7 is indicated by a white arrow on both maps.

There were more non-parasitoid insects than parasitoids collected on the outside of the pollinator hotel or while flying around the hotel (“No Material”) (Fig 5). There were also more total insects observed in bamboo clusters than in logs, but sample size was too small to make further inferences about relative insect abundances within the other material groups (Fig 5). This survey found that parasitoid insects were widespread across all material types in the pollinator hotel (Fig 5).

**Fig 5:**
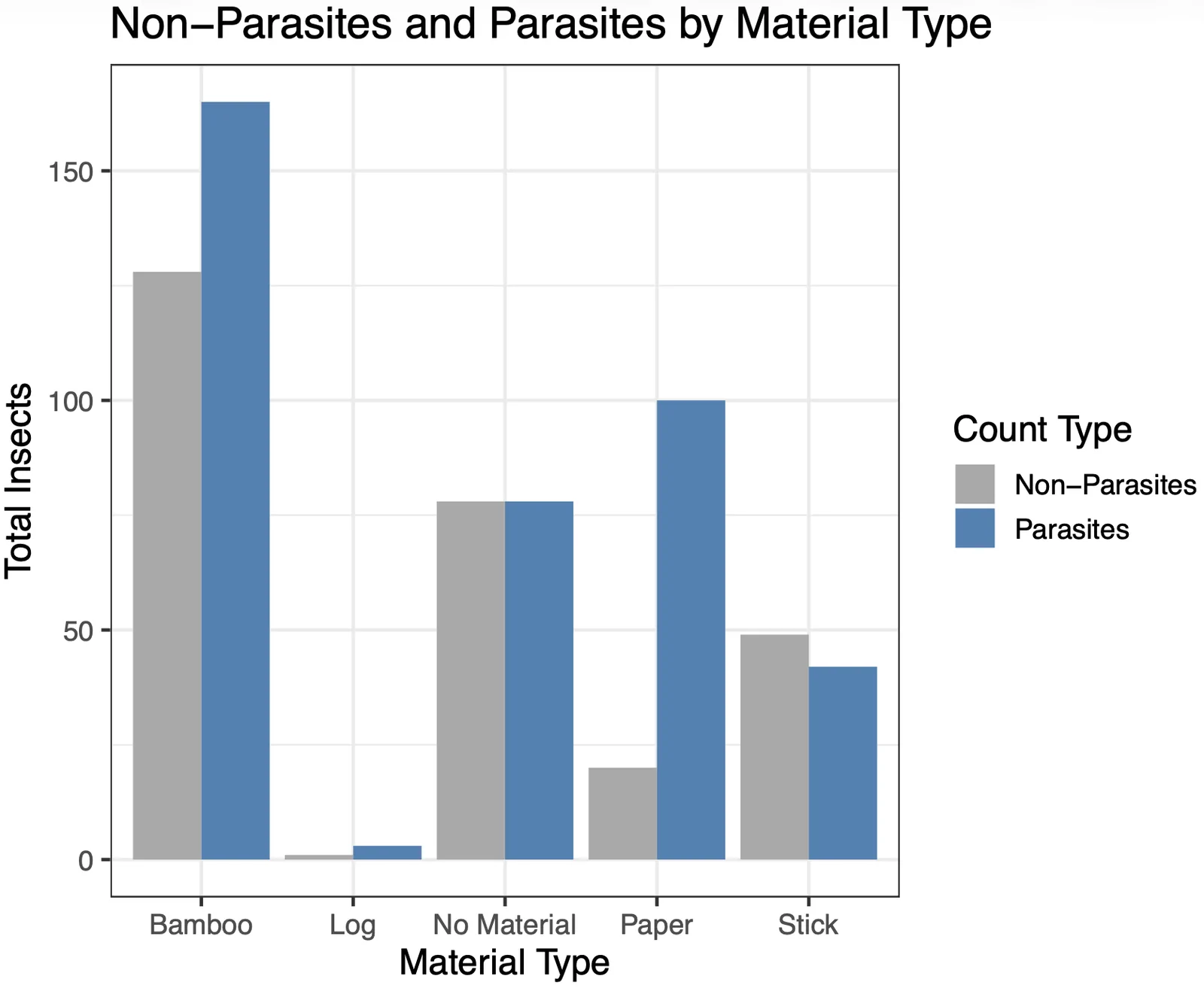
Parasitoid and non-parasitoid insects by material type. Total numbers of non-parasitic (grey) and parasitic (blue) insects found in each of the five material types examined. Insects labeled as “no material” were collected while either perched on the outside of the nest materials in a particular box or flying from box to box.

### Study 2: Parasitic contamination and distribution

Three families of parasitic Hymenoptera were observed: Ichneumonidae, Eulophidae, and Torymidae. Three individuals from the kleptoparasitic Houdini fly (Diptera: *Cacoxenus*) were also observed. Because of occurence rates, both the *Cacoxenus* samples and the two occurences of Ichneumonid wasps could not be statistically evaluated. Results are therefore focused on the families Eulophidae and Torymidae. Eulophid burden was significantly different across all three material types examined (Paper/bamboo: SE = 0.0299, z=−11.445, *p* < 0.0001. Paper/reed: SE = 1.2600, *z* = 25.783, *p* < 0.0001. Bamboo/reed: SE = 0.4770, *z* = 7.233, *p* < 0.0001) (Fig 6).

**Fig 6:**
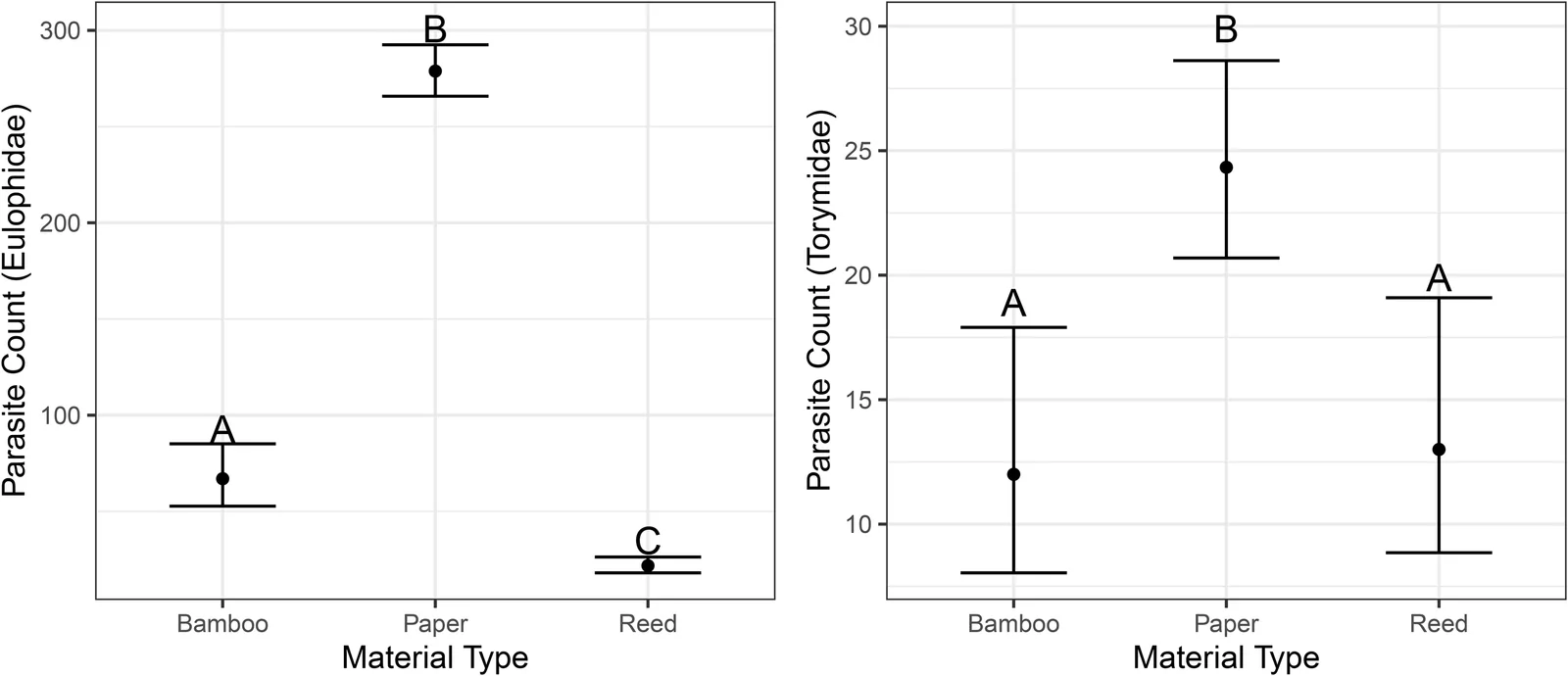
Parasitoid wasp burden by material type. Mean and 95% confidence interval plot of eulophid wasp burden (left) and torymid wasp burden (right) across all three material types. Nest materials sharing the same letter designation were not significantly different in a Tukey test.

Eulophid burden was highest in paper tubes, lowest in reeds, and intermediate in bamboo (Fig 6). Paper tubes had more than six times more eulophids observed than the other two material types, and this trend was still observed when a large statistical outlier of over 1,500 individual eulophid wasps (originating from the paper tubes taken from the UNC Asheville pollinator hotel in August) was removed from the analysis. There were no obvious differences in the August samples that led to the outlier; rather, there was simply a dramatic increase in the quantity of Eulophids emerging in that well plate. Torymid wasp burden was also highest in paper tubes (Bamboo/paper: SE = 0.1090, z=−3.210, *p* = 0.0038. Paper/reed: SE = 0.3980, *z* = 2.945, *p* = 0.0091) (Fig 6). However, there was no significant difference observed between Torymid wasp burden in bamboo versus reeds (SE = 0.2610, z=−0.283, *p* = 0.9569) (Fig 6).

The number of Eulophid and Torymid wasps also varied widely by their hotel of origin. Eulophid wasps appeared in far greater numbers at the UNC Asheville pollinator hotel than any of the other three hotels (SE = 0.00364, z=−28.362, *p* < 0.0001) (Fig 7). Eulophid wasps were also significantly more prevalent at the Centennial and Kilgore hotels than at the Court of the Carolinas hotel (Centennial: SE = 6.47000, *z* = 4.917, *p* < 0.0001. Kilgore: SE = 0.02300, z=−6.029, *p* < 0.001), and more prevalent at the Kilgore hotel than the Centennial hotel (SE = 0.09920, z=−3.279, *p* = 0.0057) (Fig 7). These trends held even when the Eulophid wasp outlier (UNCA, paper tubes, August) was removed. Torymid wasps displayed nearly the exact opposite pattern. Torymid wasps were significantly less prevalent at the UNC Asheville hotel than any of the other hotels (Centennial: SE = 8.64000, *z* = 8.827, *p* < 0.0001. Court of the Carolinas: SE = 5.90000, *z* = 8.192, *p* < 0.0001. Kilgore: SE = 1.63000, *z* = 3.396, *p* = 0.0038). The Kilgore hotel had the second lowest levels of Torymidae present. The Torymid burden at the Kilgore hotel was significantly lower than either the Centennial or Court of the Carolinas hotels (Centennial: SE = 1.63000, *z* = 6.583, *p* < 0.0001. Court of the Carolinas: SE = 1.08000, *z* = 5.717, *p* < 0.0001). Torymid burdens were not significantly different between the Centennial and Court of the Carolinas hotels (SE = 0.23200, *z* = 2.077, *p* = 0.1606).

**Fig 7:**
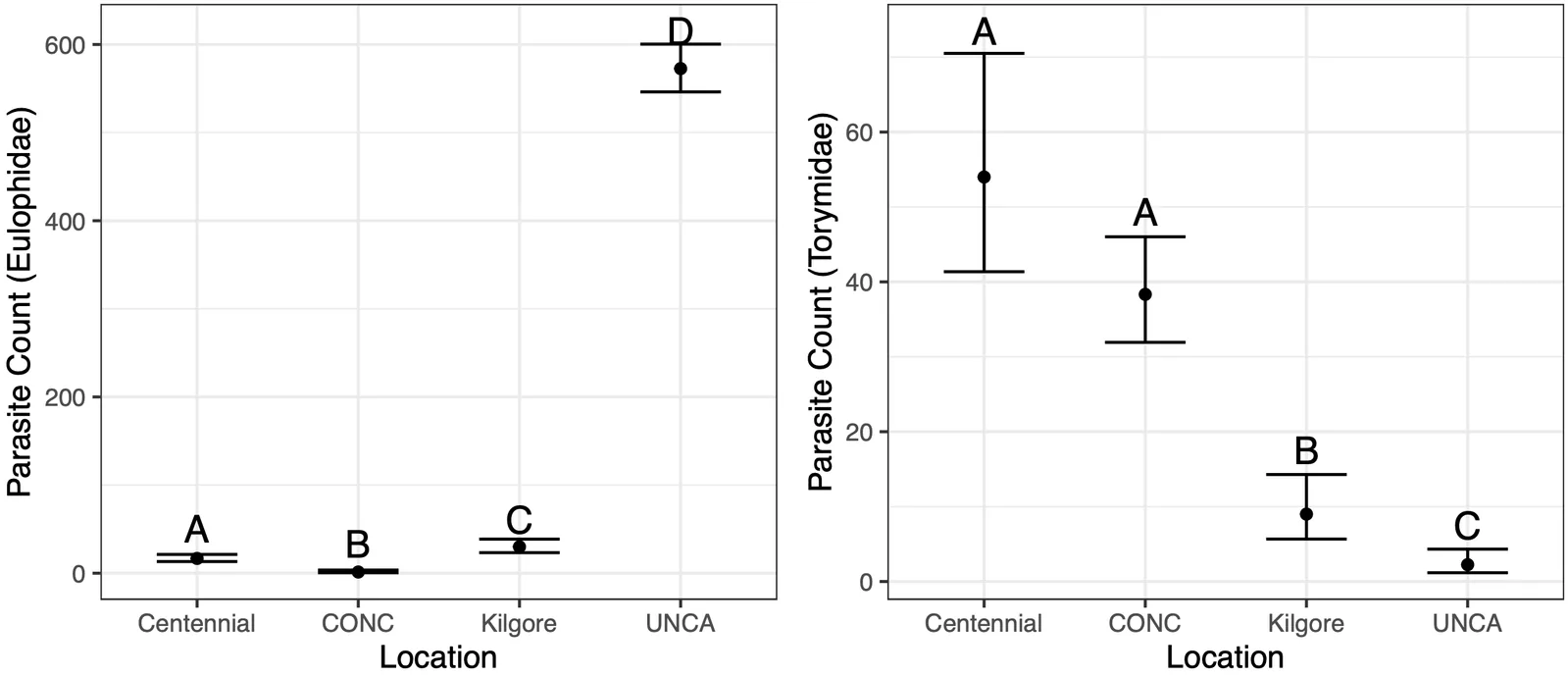
Parasitoid wasp burden by pollinator hotel. Mean and confidence interval plot of eulophid wasp burden (left) and torymid wasp burden (right) at all four pollinator hotels studied. Hotels sharing the same letter designation (within a panel) were not significantly different in Tukey tests.

### Study 3: Emergence boxes

There were no significant differences in the number of parasitoid holes present at the start of the study with the initial three groups (Control, Valve, and Rearing) (Estimate = 0.08375, SE = 0.17759, *z* = 9.425, *p* = 0.6372). However, after the separation of the Half-Valve group, Half-Valve emergence boxes had significantly more parasitoid holes at the beginning of the study (Estimate = 0.33103, SE = 0.16423, *z* = 2.016, *p* = 0.0438) (Fig 8). Because this group consisted of only two boxes and was not part of the original experimental design, this difference should be interpreted cautiously. We include this result to highlight that the boxes which lost valves mid-study may have differed initially in parasitoid load, which could have influenced subsequent outcomes.

**Fig 8:**
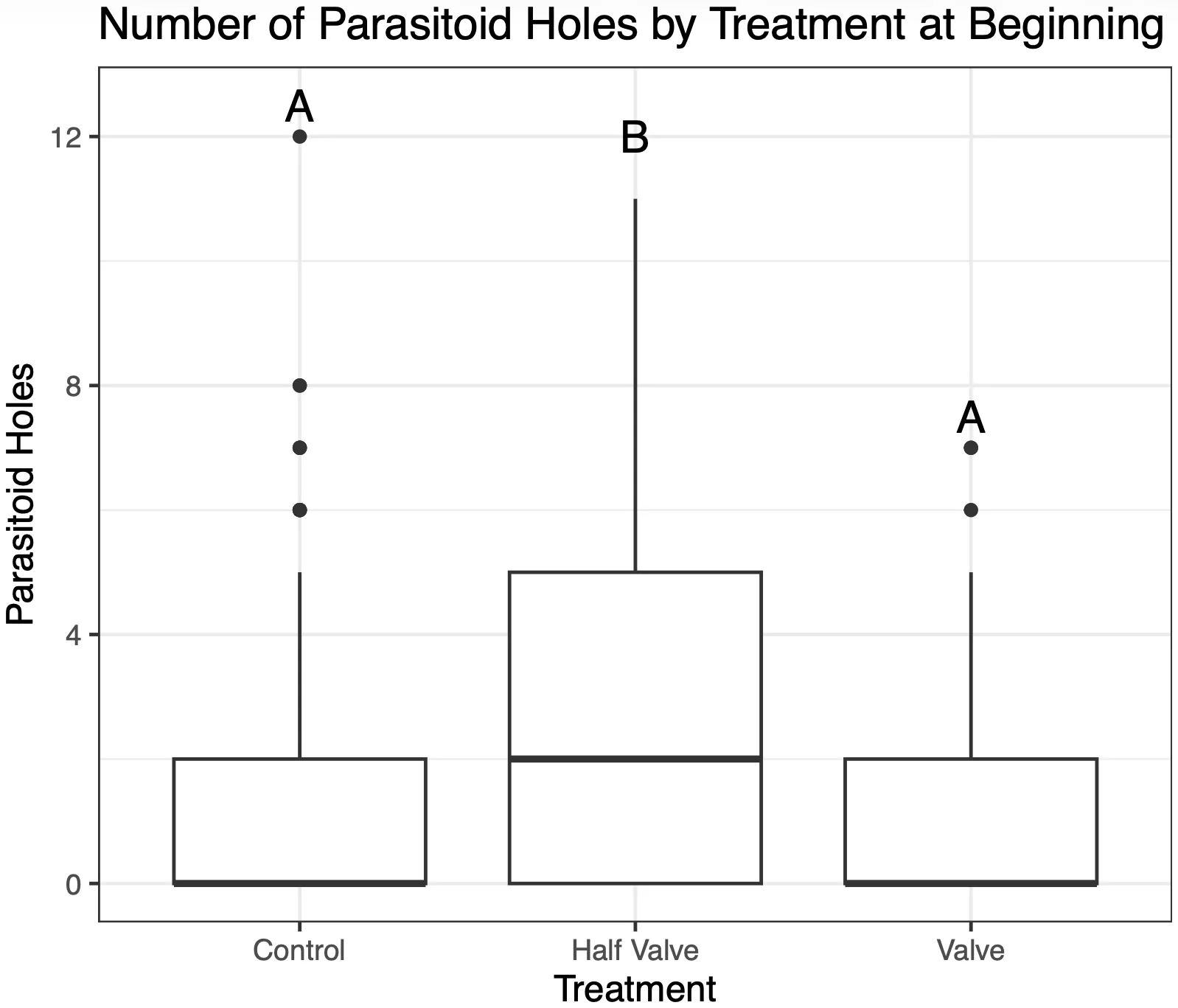
Parasitoid holes per nest tube by treatment group at study start. Nest materials in the half valve treatment group had significantly more parasitoid holes than the other three treatments.

There were no significant differences between the treatment groups in the number of additional parasitoid holes observed at the end of the study, either when the two Half-Valve boxes were separated as their own treatment (Estimate =−0.2464, SE = 0.6417, z=−0.465, *p* = 0.6661) (Fig 9) or when combined into a single Valve treatment (Estimate = 0.14860, SE = 0.14781, *z* = 1.005, *p* = 0.314745). However, when separated, the Half-Valve group consisted of only two boxes, limiting statistical power and the ability to detect meaningful differences among treatments. Diameter was also not a signficant factor in the number of parasitoid holes either at the start (Estimate =−0.02574, SE = 0.04215, z=−0.611, *p* = 0.541346) or end of the study (Estimate =−0.04662, SE = 0.04676, z=−0.997, *p* = 0.3188). Extremely low levels of additional parasitoid holes were observed after a year in the emergence boxes across all three emergence box treatments, with the vast majority of nest materials gaining no additional parasitoid holes (Fig 9).

**Fig 9:**
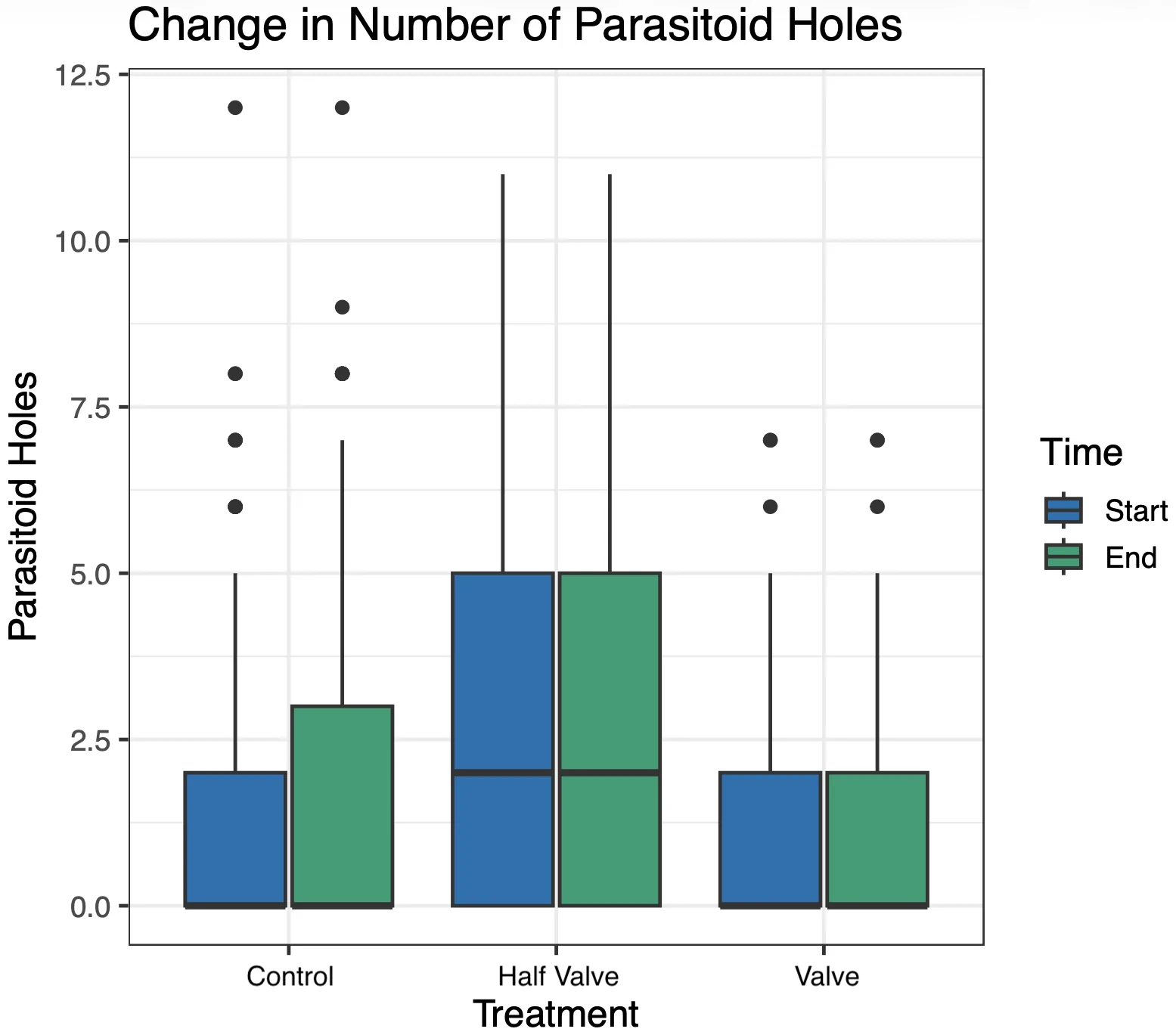
Parasitoid holes per nest tube at start and end of experiment. Box and whisker plot of the number of parasitoid holes at the start (blue) and end (green) of the experiment across all three experimental treatments.

The proportion of nest materials with evidence of re-nesting was low across all three treatments (Fig 10). For this study, re-nesting was defined by a change in the material of the cap or a shift from the presence of a hole to an intact cap. Out of 261 total observations, there were 41 instances of re-nesting recorded, and every material type had at least once instance of re-nesting(Fig 11). Of these 41 instances, 21 were transitions between different types of mud cap(Fig 11). We cannot ignore the possibility that changes in mud color reflect aging or oxidation of the mud rather than a true re-nesting event. When mud color shifts are included, the Control and Valve treatments both had a re-nesting proportion of approximately 0.13. The Half-Valve treatment had significantly lower levels (Estimate =−1.5563, SE = 0.7625, z=−2.041, *p* = 0.0413). However, when mud color changes are excluded, there are no significant differences between the three treatments (Estimate =−1.06582, SE = 1.09151, z=−0.976, *p* = 0.329) (Fig 10). Additionally, with the exlusion of mud color changes, the proportion of renesting events drops to between 0.164 and 0.462.

**Fig 10:**
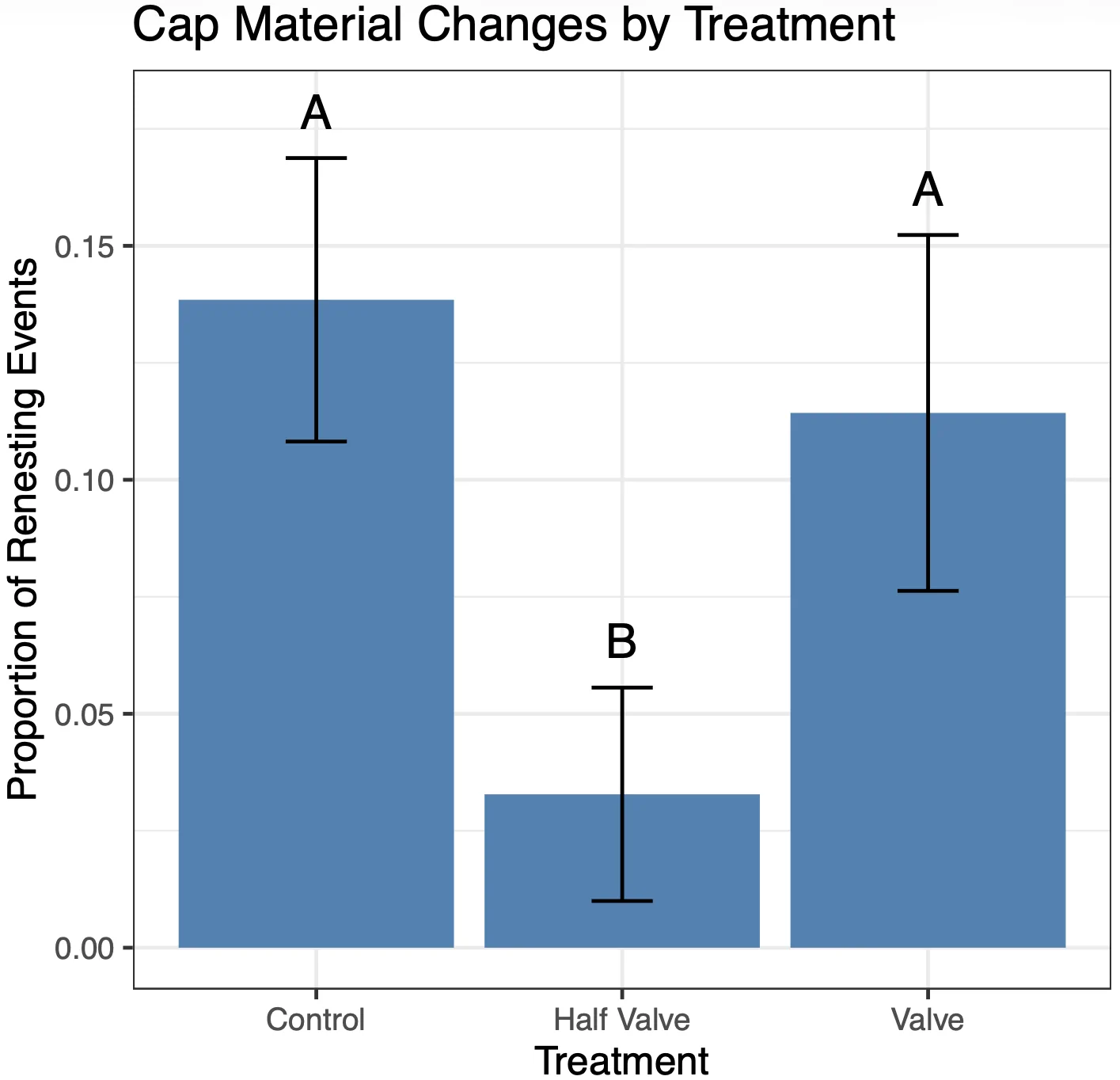
Re-nesting proportion by treatment. Proportion of nest materials that experienced a change in cap material or a change in the presence of a hole in the nest cap indicative of re-nesting over the course of a year in the emergence boxes. Confidence intervals are also shown. Transitions between different types of mud have been removed.

**Fig 11:**
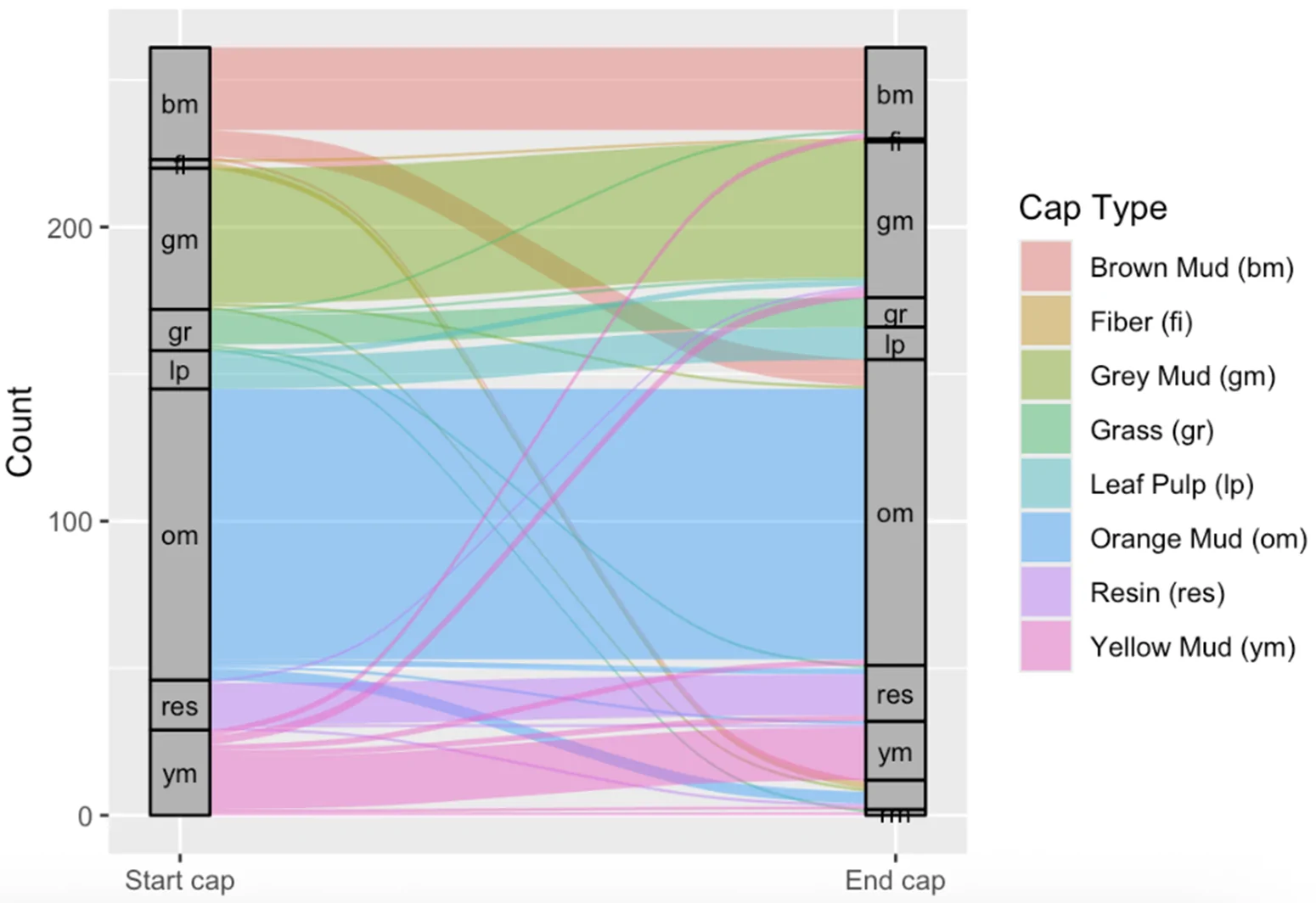
Cap material transitions. Sankey diagram of cap material changes from the start to the end of the emergence box study.

There were no statistical differences in the number of re-nesting events that occurred at the four different pollinator hotels either including mud cap transitions ($\chi^{2}=5.3158$, df = 3, *p* = 0.1501) or excluding them ($\chi^{2}=2.4343$, df = 3, *p* = 0.7883). The proportion of re-nesting events was also not tied to a particular material type either including mud cap transitions($\chi^{2}=1.0534$, df = 3, *p* = 0.7883) or excluding them ($\chi^{2}=2.5798$, df = 3, *p* = 0.461).

## Discussion

The results of the biodiversity survey (Study 1) show that the UNC Asheville pollinator hotel is home to a wide variety of bee and wasp species, but that diversity is unevenly distributed across the hotel. The numbers of observed insects were distributed unevenly throughout the twelve sub-boxes in the hotel as indicated by a chi-square test of independence, but there were no trends across rows or columns of sub-boxes. Additionally, there were no significant differences in the number of families across different areas of the hotel. This was an unexpected result because the upper row of the hotel (sub-boxes 1-4) are shaded by the roof for several hours after the bottom rows have begun to receive sun. Orienting pollinator hotels towards the southeast is standard practice in North America and Europe, and nests with exposure to the morning sun are thought to be more attractive to bees [16], but in our study, the differences in sun exposure appeared to have little affect on overall trends in insect or family abundance.

These results also indicate that parasites are indeed very common in the pollinator hotel. Several of the material types surveyed in Study 1, including paper tubes and bamboo, had nearly the same number of parasitoid insects as non-parasitoid insects found inside. Previous research has found that paper tubes are particularly vulnerable to parasitoids, especially the parasitic wasp *Monodontomerus obscurus* [17]. Beyond parasitic wasps, birds and rodents have also been observed dismantling bee nests housed in paper tubes, consuming the larvae, and using the paper itself for their own nests [17]. Bamboo and reeds have previously been considered safer for bees, and the thicker walls of these materials can offer some protection from other common parasites like *Leucospis affinis* [16]. However, the high presence of parasitoids in these materials in the biodiversity survey indicates an underlying problem. Before the start of this research in 2022, the UNC Asheville pollinator hotel had not been properly maintained or cleaned since its construction in 2016. Regular maintenance is necessary to prevent the buildup of parasites and diseases in pollinator hotels [9], and the absence of this maintenance likely explains the large number of parasites observed. These results, while striking, likely overestimate the number of parasitoid insects present in the pollinator hotel, as only adult insects were included in the data set. Unparasitized cocoons containing healthy larvae were therefore likely present but not counted. Nevertheless, the high prevalence of parasites was corroborated in Study 2, where insects were reared from nests rather than relying on a snapshot of adult presence.

The four families of parasites represented in Study 2 differed from those documented in the first study. Ichnumonid and Eulophid wasps were not documented in the first study at all (likely due to small size in the case of the Eulophids), while significant levels of *L. affinis* wasps and chrysidid were observed that were not then documented in the second study. Leucospid and chrysidid wasps were observed again in the emergence box study (Study 3), though in low numbers (one individual each). The discrepancies in the identified families between the three studies could be due in part to the sampling method. It could also be due to the confluence of factors contributing to nesting success – flight range, proximity and co-occurrence of resources, and other factors. The second study used only nests made in new, undamaged nest materials that had only been in the pollinator hotel for two months. It is possible that leucospids and chrysidids are more prone to parasitizing damaged materials, or that the positioning of the new tubes in tight bundles made it more difficult for the wasps to penetrate them. Leucospid wasps, specifically, rely on ovipositors to penetrate up to 3/4 of an inch into the outside of a nest to reach the bee’s cocoon [16]. The materials located towards the inside of our material bundles would likely have been inaccessible to the wasps, which may have influenced the types of parasites seen in the study.

The patterns of parasite burden observed across the eulophid and torymid species were more expected. The far higher abundance of parasites observed in paper tubes as compared to bamboo and reeds fits both with our hypotheses and with previous research that paper tubes are more susceptible to parasitism [17]. Eulophid wasps were also more common in bamboo nests than reed ones. However, as there was no significant difference between the levels of Torymid wasps present in reeds versus bamboo, we are unable to fully support our original hypothesis that reeds are more susceptible to parasitism than bamboo. The susceptibility of bamboo to parasitoids in general is much less well studied than the susceptibility of paper tubes. Further, we are prevented from making determinations about the reasons behind the greater burden of eulophid wasps in bamboo tubes by the obscure nature of the wasp itself. The species of wasp observed at the pollinator hotel was identified as belonging to the family Eulophidae, genus *Melittobia*, by the Plant Disease and Insect Clinic (NC State University). However, the species was unable to be determined, and the life history and host(s) of this wasp are unknown. Future research should also include the effects of diameter as a covariate factor that may affect overall parasite abundance in addition to material type.

The patterns of parasite burden across the four pollinator hotel locations were less expected. Torymid and eulophid parasites displayed nearly opposite trends. The levels of eulophid parasites were orders of magnitude lower in the three NC State hotels than the UNC Asheville hotel. Torymid wasps (specifically *Monodontomerus obscurus*) which occurred in significantly greater numbers in all three NC State pollinator hotels, are known parasitoids of *Osmia* and *Megachile* bees [17]. Parasitoid wasps have been suggested to be especially susceptible to host competition because of their reliance on limited hosts to complete their reproductive cycles [47]. While both *M. obscurus* and many wasps in the eulophid genus *Melittobia* are generalists, and thus able to parasitize a variety of host species [48–50], the limited number of hosts within the pollinator hotel may still lead to interspecific competition. Additionally, since the hosts are more concentrated in the pollinator hotel than they would usually be in the wild, the wasps may have reduced barriers to finding hosts [47]. Many species of wasps have been shown to co-parasitize the same larva, even if only one species can ultimately develop [51]. If the torymid and eulophid wasps were engaging in co-parasitism or subjected to increased interspecific competition due to the increased population density of the pollinator hotel environment, competitive exclusion of torymids by the eulophids may have occured at the UNC Asheville hotel.

Varying population levels of suitable hosts may also be the distinguishing factor between the differing burden of torymid wasp parasites among the three NC State hotels. Identification of insects from the parasitoid study was only conducted at the family level, but if genus or species-level identifications were made, the three hotels would likely have differing population levels of susceptible bees. The populations of the bees themselves were likely affected by the differing environments of the hotels themselves; while all three NC State pollinator hotels were roughly the same size and contained a similar spread of material types and diameters, the environments and flowers provided close to the hotel differed widely among the three locations (Table 1). While information was gathered on major flowering plant species observed near the pollinator hotels when nest material bundles were collected (April, June, and August), there is no comprehensive record of flower species or abundance near the pollinator hotels over the course of these studies. The availability or abundance of particular types of flowers may have impacted the species of pollinators that chose to make their homes within the pollinator hotel. This is because different flowers vary in both their attractiveness to pollinators (due to color or morphology) and their nutritional content [52]. Both factors may influence pollinator hotel community dynamics, which may in turn have influenced the parasitoid burden and which parasitic wasp species took up residence in the hotel. Overall, the results of the second study suggest that both environmental differences among hotel sites and competition among parasitoid species jointly influenced the observed variation in parasitoid prevalence across pollinator hotels. Future studies that examine specific environmental conditions, including pollinator hotel size and nearby floral resources, are necessary to illuminate this relationship further.

One of the primary concerns with emergence boxes as a tool to reduce parasite burden was the risk of confining healthy, unparasitized nest cells in the same small space as parasitized cocoons. Because parasitoids such as *Monodontomerus* have shorter generation times than their hosts, they can multiply greatly over a single season in a bee hotel or emergence box. If the parasitoids were able to hatch before most of the bees, the box itself might serve as an accelerator of parasitism, since it increases population density even more than in the hotels itself. The increased population density of pollinator hotels has been previously linked to increased risk of parasitism [26]. However, small numbers of new parasitoid holes were observed across all three treatments at the end of the emergence box study, which indicates that parasitoid wasps did not eclose and parasitize bee cocoons before the bees themselves were able to leave the boxes. While some nest tubes did increase parasitoid holes over the study, the data were heavily skewed towards zero levels, indicating that additional parasitism inside the emergence boxes was uncommon. This bodes well for the success of emergence boxes as a maintenance tool.

The other main concern with emergence boxes is the potential for bees to re-nest inside them. While an emergence box is in use, the rest of the materials in the hotel can be cleaned and replaced as needed to reduce the buildup of parasitoid wasps, mites, and fungal pathogens [9]. Cavity-nesting bees seek protected cavities to nest in, and, if the bee has just emerged from an emergence box, it may decide to return to the box to make its new nest. In the third study, while the proportion of bees that re-nested in the emergence box was not zero, it was low (at or below 12.5% when mud type changes are included, at or below 4.6% when mud type changes are not included) across all three treatments. This indicates that the great majority of bees that hatch out of the emergence boxes found another area (presumably the cleaned and refreshed pollinator hotel nearby) to make their nests in. Most of these re-nesting events consisted of transitions between different types of mud caps, rather than transitions from resin, leaf pulp, or grass. This indicates that mason bees, rather than leafcutter bees, resin bees, or grass wasps, likely return to nest in the emergence boxes most frequently. However, because the mud color changes could potentially be due to environmental effects and because every type of material underwent at least one re-nesting event, no definitive conclusions can be drawn about which families of bees are most likely to re-nest in an emergence box. Additionally, because insects were identified only to the family level and because of the relatively small number of nest materials that were reared out in the lab, we were unable to make concrete connections between particular groups of bees and their relative likelihood to re-nest in the emergence boxes. Insects were unable to be further identified to genera and/or species due to the damage samples sustained while in storage from sustained power outages associated with Hurricane Helene.

Future studies should focus on this re-nesting phenomenon and whether particular families, genera, or species of bees are more likely than others to return to nest in the emergence boxes. Given these data, the risk of installing an emergence box could be evaluated on a case-by-case basis, based on the composition of that hotel’s bee population.

Surprisingly, the attachment of a one-way valve apparatus to the entrance of the hotel resulted in no obvious reduction in the proportion of bees that re-nested in the hotel. The treatment that showed the lowest rate of re-nesting was the half-valve treatment. While this could be due to an initial preventative effect from the one-way valve, the lack of any difference between the re-nesting rates of the control and valve treatment groups suggests other factors may be in play. Open-air nest sites, such as the nearby pollinator hotels, may have been more attractive to the bees than re-entering the closed space of the emergence boxes. The half-valve group also had significantly more parasitoid holes at the beginning of the study than the other two groups, indicating that these boxes contained more parasitized nests than the other treatments. Fewer viable nests may have led to fewer bees hatching from these boxes, and therefore fewer bees returned to re-nest in the emergence boxes. Because half of the emergence boxes equipped with the one-way valve were later reassigned to the half-valve section (due to the valve falling off), the sample size for this part of the study was quite low. Additional tests of the one-way valve as a re-nesting preventative would be useful to further illuminate whether or not the valve provides meaningful deterrence.

Taken together, the results of these three studies highlight both the ecological complexity and management challenges of artificial nesting habitats for solitary bees. Patterns of biodiversity and parasitism at the UNC Asheville pollinator hotel reveal how environmental orientation, maintenance history, and material choice shape community composition and parasite load. The differences in parasitoid prevalence and family across the four hotel locations underscore the strong influence of site-specific factors and potential interactions among parasitoid species. Finally, the emergence box experiment demonstrates that these tools may allow for pollinator hotel maintenance without significantly increasing parasitism risk or encouraging extensive re-nesting within the emergence boxes themselves. While certain behaviors, such as limited re-nesting, merit further study, the overall findings suggest that careful placement, regular cleaning, and the strategic use of emergence boxes can enhance the long-term health and sustainability of managed pollinator populations.

Our results should be used to improve the maintenance practices for pollinator hotels because of their value not only as conservation tools, but as educational platforms that can shape public understanding of native bee ecology. Pollinator hotels are ideal venues for students, citizen scientists, and the broader community to directly observe species interactions and the consequences of different management decisions. Effective pollinator hotel management contributes to sustaining native bee populations that are integral to the reproduction of both wild and managed flowering plants systems. As habitat loss and anthropogenic pressures continue to threaten pollinator communities globally, initiatives that integrate research, maintenance, and public education can help foster ecological literacy while directly supporting pollinator preservation. In this way, well-managed pollinator hotels can function not only as nesting habitat but as living classrooms that connect local conservation actions to global concerns about biodiversity, ecosystem resilience, and our agricultural system.
